# Editorial: New Insights on Bruton’s Tyrosine Kinase Inhibitors

**DOI:** 10.3389/fimmu.2021.804735

**Published:** 2021-11-23

**Authors:** C. I. Edvard Smith, Jennifer R. Brown, Rula Zain

**Affiliations:** ^1^ Biomolecular and Cellular Medicine, Clinical Research Center, Department of Laboratory Medicine, Karolinska Institutet (KI), Huddinge, Sweden; ^2^ Chronic Lymphocytic Leukemia Center, Division of Medical Oncology, Dana-Farber Cancer Institute and Harvard Medical School, MA, United States; ^3^ Department of Clinical Genetics, Center for Rare Diseases, Karolinska University Hospital, Stockholm, Sweden

**Keywords:** BTK, ibrutinib, inhibitors, leukemia, lymphoma, Waldenström’s macroglobulinemia, autoimmunity, structure-function

In 1993 Bruton’s tyrosine kinase (BTK; initially named ATK and BPK) was identified by two independent research groups. The corresponding gene, *BTK*, is mutated in X-linked agammaglobulinemia suggesting that inhibitors of this enzyme would profoundly affect B lymphocyte development. Fourteen years after the *BTK* gene cloning, the first clinically effective inhibitor of this enzyme, later named ibrutinib (imbruvica^®^) was reported. In 2013, the first publication on its utility in leukemia and lymphoma was published in the *Journal of Clinical Oncology*. The following progress in research and development based on these early findings can be described as no less than a revolution, altering the management of chronic lymphocytic leukemia, mantle cell lymphoma and Waldenström’s macroglobulinemia, and more, by replacing chemotherapy with targeted therapy.

While BTK is a central signal transducer for B lymphocyte development in both man and mouse, and is well-known to most immunologists, the dramatic events in the treatment of leukemia and lymphoma have mainly appeared in medical, hematological and oncological journals. For this reason, the editors believed that it would be timely to review the field for an immunological audience and to achieve this, 5 reviews covering various topics of BTK inhibitors (BTKi) were collected. Apart from treatment of B cell malignancies, these reviews also include autoimmune diseases, for which BTKi therapy is still in its infancy, but where it is projected that they will have a profound impact. There is also a description of structure-function relationships among the increasing number of clinically developed BTKi.

Ahn and Brown insightfully describe many aspects of optimized BTK targeting in a clinical setting. This includes a comparison between different clinically available compounds, and they summarize in table format the key studies that have contributed to the current use of these drugs. They also elaborate on safety and drug resistance and how to overcome acquired unresponsiveness. Adverse effects include bleeding, hypertension, atrial fibrillation and opportunistic infections. They find that the ongoing randomized trials will provide a better distinction of both safety and efficacy. They also conclude that combinatorial treatments, including the use of BTKi together with apoptosis-modifying therapies, such as inhibition of BCL-2, can achieve uniquely deep responses. As it is still early days in the development of combinatorial treatments, there remains room to tackle and improve this concept from a basic immunology standpoint.

Smith and Burger take a deep look at the mechanisms underlying both responsiveness and resistance to BTKi and put this into perspective by careful analysis of where they interfere in the B cell receptor (BCR) signaling pathway. They also compare the *primary* mutations predisposing and underlying the development of chronic lymphocytic leukemia and Waldenström’s macroglobulinemia with both the BTKi resistance patterns and the genetic defects causing primary immunodeficiencies. In this analysis they identify inconsistencies that cannot readily be explained, and they conclude that: “The common linear representation of the BCR-signaling pathway, which trifurcates into the NF-κB, NFAT and AP-1 arms, does not fully explain the observed pattern of resistance mutations in patients treated with BTKi.” This could represent a starting point for immunological experimentalists to undertake the challenge of resolving this enigma.

Palma et al. concentrate their efforts on chronic lymphocytic leukemia and the immunomodulatory effects of BTKi. They describe the impact on both innate and adaptive immunity, including how different BTKi affect various lymphocyte and other leukocyte subsets during treatment. They go on to discuss the role of BTKi in the susceptibility to infections, which is another adverse effect of great importance for clinical management, and with implications for basic immunology. Finally, they touch upon the effect of BTKi for vaccine responses, which is a topic of particular interest in the time of a pandemic. BTKi do reduce humoral immunity to vaccines. While there was initial hope that BTKi may ameliorate Covid-19, the data thus far have not borne that out.

Zain and Vihinen bring up another important aspect, given the currently increasing number of BTKi, namely structure-function relationships. In a pedagogic way they explain the conformation of some of the domains of BTK with special emphasis on the catalytic (kinase) entity. This review can also be used as a general introduction to the field of kinases, which are such important regulators in lymphocytes. The reader will learn about the three-dimensional structure of the ATP-binding pocket and how inhibitors, both irreversible (those forming a covalent bond with a critical cysteine) and reversible (which have a different binding mode), will tether to this site. A crucial aspect for the understanding of adverse effects is how specificity can be obtained, and why certain BTKi show more cross-reactivity.

Ringheim et al. review the recent developments in the use of BTKi for the treatment of autoimmunity. While irreversible BTKi have dominated the field of B cell malignancies, pioneered by ibrutinib, reversibly-binding BTKi have preferentially been used in the recent clinical studies of autoimmunity. Owing to the selective effect of BTKi on B lymphocytes, treatment of autoimmune disorders can also generate basic information on the origin of the cells causing disease. Ringheim et al. go on to compare all the different BTKi that have been used. They also report on recent studies on multiple sclerosis and pemphigus vulgaris, for which they conclude that BTKi treatment is most promising, whereas the verdict is still out for rheumatoid arthritis.

It is our hope that this Research Topic, not only can make immunologists aware of the significant progress that has taken place in the field of BTKi, but that it can also serve as an inspiration for using BTKi in the experimental setting to find solutions to unresolved immunological problems. Moreover, while BTKi may preferentially be of interest to B lymphocyte biology, given the intricate relationship between different leukocyte subsets, the knowledge obtained from studying the effects of BTKi can certainly extend far beyond this lineage.

## Author Contributions

All authors listed have made a substantial, direct, and intellectual contribution to the work and approved it for publication.

## Funding

This work was supported by the Swedish Cancer Society, Stockholm County Council (ALF-project) and Swedish Medical Research Council.

## Conflict of Interest

JB has served as a consultant for Abbvie, Acerta/Astra-Zeneca, Beigene, Bristol-Myers Squibb/Juno/Celgene, Catapult, Genentech/Roche, Eli Lilly, Janssen, MEI Pharma, Morphosys AG, Nextcea, Novartis, Pfizer, Rigel; received research funding from Gilead, Loxo/Lilly, Verastem/SecuraBio, Sun, TG Therapeutics; and served on the data safety monitoring committee for Invectys.

The remaining authors declare that the research was conducted in the absence of any commercial or financial relationships that could be construed as a potential conflict of interest.

## Publisher’s Note

All claims expressed in this article are solely those of the authors and do not necessarily represent those of their affiliated organizations, or those of the publisher, the editors and the reviewers. Any product that may be evaluated in this article, or claim that may be made by its manufacturer, is not guaranteed or endorsed by the publisher.

